# An oxidized abasic lesion inhibits base excision repair leading to DNA strand breaks in a trinucleotide repeat tract

**DOI:** 10.1371/journal.pone.0192148

**Published:** 2018-02-01

**Authors:** Jill M. Beaver, Yanhao Lai, Shantell J. Rolle, Liwei Weng, Marc M. Greenberg, Yuan Liu

**Affiliations:** 1 Biochemistry Ph.D. Program, Florida International University, Miami, FL, United States of America; 2 Department of Chemistry and Biochemistry, Florida International University, Miami, FL, United States of America; 3 Department of Chemistry, Johns Hopkins University, Baltimore, MD, United States of America; 4 Biomolecular Sciences Institute, School of Integrated Sciences and Humanities, Florida International University, Miami, FL, United States of America; University of South Alabama Mitchell Cancer Institute, UNITED STATES

## Abstract

Oxidative DNA damage and base excision repair (BER) play important roles in modulating trinucleotide repeat (TNR) instability that is associated with human neurodegenerative diseases and cancer. We have reported that BER of base lesions can lead to TNR instability. However, it is unknown if modifications of the sugar in an abasic lesion modulate TNR instability. In this study, we characterized the effects of the oxidized sugar, 5’-(2-phosphoryl-1,4-dioxobutane)(DOB) in CAG repeat tracts on the activities of key BER enzymes, as well as on repeat instability. We found that DOB crosslinked with DNA polymerase β and inhibited its synthesis activity in CAG repeat tracts. Surprisingly, we found that DOB also formed crosslinks with DNA ligase I and inhibited its ligation activity, thereby reducing the efficiency of BER. This subsequently resulted in the accumulation of DNA strand breaks in a CAG repeat tract. Our study provides important new insights into the adverse effects of an oxidized abasic lesion on BER and suggests a potential alternate repair pathway through which an oxidized abasic lesion may modulate TNR instability.

## Introduction

Trinucleotide repeat (TNR) expansions are associated with over 40 human neurodegenerative diseases, including Huntington’s disease (CAG/CTG), Friedreich’s ataxia (GAA/TTC), fragile X syndrome (CGG/CCG), and myotonic dystrophy (CTG/CAG) [1, 2]. TNR instability results from the formation of secondary structures such as hairpins, triplexes, quadruplexes, and sticky DNA [3–5] during DNA replication, repair, recombination, and gene transcription [3, 6–8]. Base excision repair (BER) within TNR tracts plays a critical role in modulating repeat instability [9–11], which results from a loss of coordination between key BER proteins and cofactors [4, 9, 12–18]. The formation of hairpins in a TNR tract can block 5’-flap cleavage by flap endonuclease 1 (FEN1), resulting in alternate flap cleavage in which FEN1 removes fewer nucleotides [13]. We have previously demonstrated that a DNA base lesion that occurs in a TNR tract can lead to the maintenance of stability or expansion/deletion of TNRs via either the short-patch/single-nucleotide BER, or long-patch BER sub-pathway [11–13, 18, 19]. If a base lesion is repaired by short-patch/single-nucleotide BER, the dRP lyase activity of DNA polymerase β (pol β) removes a 5’-deoxyribose phosphate at the 5’-side of a 1 nt-gap. The pol β polymerase activity subsequently fills in the gap to generate a nick that is sealed by DNA ligase. In this case, only one nucleotide is synthesized, thereby leading to the maintenance of TNR stability. However, if TNRs slip out from their templates resulting in the formation of TNR hairpins on the damaged strand or template strand and multi-nucleotide gaps, this forces the repair into the long-patch BER subpathway, in which pol β performs multi-nucleotide gap-filling synthesis and strand-displacement synthesis to synthesize additional units of TNRs causing TNR expansion/deletion [11–13, 18]. We further demonstrated that when the addition of nucleotides by pol β exceeds the number of nucleotides removed by FEN1, repeat expansion occurs [12, 13]. A recent study from our group showed that the addition of nucleotides by pol β can be stimulated by a crosstalk between the polymerase and a mismatch repair protein complex, MSH2-MSH3, resulting in TNR expansion [20]. Alternatively, when hairpins form in the template strand of a TNR tract, pol β can skip over the hairpin, leading to addition of fewer nucleotides than those removed by FEN1 cleavage, and resulting in repeat deletion [18, 21]. Since the tandem purines in TNR tracts are hotspots for oxidative DNA damage [22], repeated cycles of oxidative damage and DNA repair can lead to an accumulation of deletions and expansions through a “toxic oxidation cycle” [2, 9, 23]. We have reported that oxidized and alkylated DNA bases can result in TNR instability, and that the location of a base lesion in a duplex TNR tract determines the type of repeat instability [12, 18, 24]. A base lesion at the 5’-end of a TNR tract induces expansions as a result of the formation of a stable hairpin in the downstream region of the damaged strand, while a lesion in the middle of the repeat tract induces deletions as a result of the formation of a hairpin in the template strand [12]. However, on a TNR hairpin, guanines located in the single-stranded loop of a TNR hairpin are more susceptible to the formation of an oxidized base lesion, 8-oxo-guanine (8-oxodG) [22, 23]. Moreover, an 8-oxodG formed in the stem region or adjacent to a hairpin is preferentially relocated into the loop region [25]. Interestingly, BER of an 8-oxodG in the loop of a TNR hairpin results in hairpin removal and the prevention of TNR expansions [26, 27]. This is accomplished by the formation of a double-flap intermediate following AP endonuclease 1 (APE1) 5’-incision of the abasic site in the hairpin loop, and the coordination between FEN1 and the 3’-5’ exonuclease activity of APE1 or Mus81/Eme1, which removes the 5’- and 3’-flaps, respectively [26, 27]. In addition, we have reported that an oxidative DNA lesion, 5', 8-cyclo-2'-deoxypurine, in the template strand of a TNR tract induces the formation of a small loop that is subsequently skipped over by pol β during BER leading to TNR deletion [21]. Thus, the location and type of a base lesion play determining roles in governing TNR instability via BER.

However, DNA bases are not the only targets modified when genomic DNA is attacked by DNA damaging agents. Several oxidized abasic sites are produced via a variety of oxidative pathways and agents [28]. An oxidized abasic site presents a challenge to DNA replication, as well as BER. Oxidized sugars inactivate pol β [29] and a bacterial glycosylase [30]. However, it is unknown whether oxidized abasic sites adversely affect DNA repair efficiency and/or TNR instability during BER given the fact that the tandem purine repeats in TNR tracts are hotspots of oxidative DNA damage and formation of oxidized sugars. In this study, we explored whether formation of the oxidized abasic site, 5’-(2-phosphoryl-1,4-dioxobutane) (DOB) affects repeat instability during BER by modulating BER protein activity and efficiency. By employing oligonucleotide substrates that mimic the intermediates generated during BER in a CAG repeat duplex and small CAG hairpin containing either a DOB, a native abasic site (AP), or a chemically stabilized, reduced abasic site analogue (THF), we found that the DOB lesion greatly inhibited pol β synthesis activity. Inhibition was ascribed to crosslink between DOB and pol β. Surprisingly, we discovered that DOB prevented formation of the repaired product by inhibiting DNA ligase I (LIG I) by crosslinking with this enzyme as well. Inhibition of these processes resulted in an accumulation of single-strand DNA (ssDNA) breaks in the repeat tracts. Thus, our study suggests that an oxidized abasic site promotes TNR instability by facilitating DNA recombination rather than directly modulating repeat instability during BER.

## Materials and methods

### Materials

Oligonucleotides containing the DOB lesion were synthesized as previously described [31]. All other DNA oligonucleotides were synthesized by Integrated DNA Technologies (IDT, Coralville, IA, USA). T4 polynucleotide kinase and terminal deoxynucleotidyltransferase were purchased from Thermo Fisher Scientific (Waltham, MA, USA). Radionucleotides [γ-^32^P] ATP (6000 mCi/mmol) and Cordycepin 5’-triphosphate 3’-[α-^32^P] (5000 mCi/mmol) were purchased from Perkin Elmer Inc. (Boston, MA, USA). Deoxynucleotide 5’-triphosphates (dNTPs) were from Fermentas (Glen Burnie, MD, USA). Micro Bio-Spin 6 chromatography columns were purchased from Bio-Rad Laboratories (Hercules, CA, USA). All other chemical reagents were purchased from Thermo Fisher Scientific (Waltham, MA, USA) and Sigma-Aldrich (St. Louis, MO, USA). Uracil-DNA glycosylase (UDG) was from New England Biolabs (Ipswich, MA). Recombinant human pol β was expressed in *Escherichia coli* and purified as described previously [13]. Pol β K72A mutant protein was a generous gift from Dr. Samuel H. Wilson at the National Institute of Environmental Health Sciences/National Institutes of Health.

### Oligonucleotide substrates

**Fig 1:**
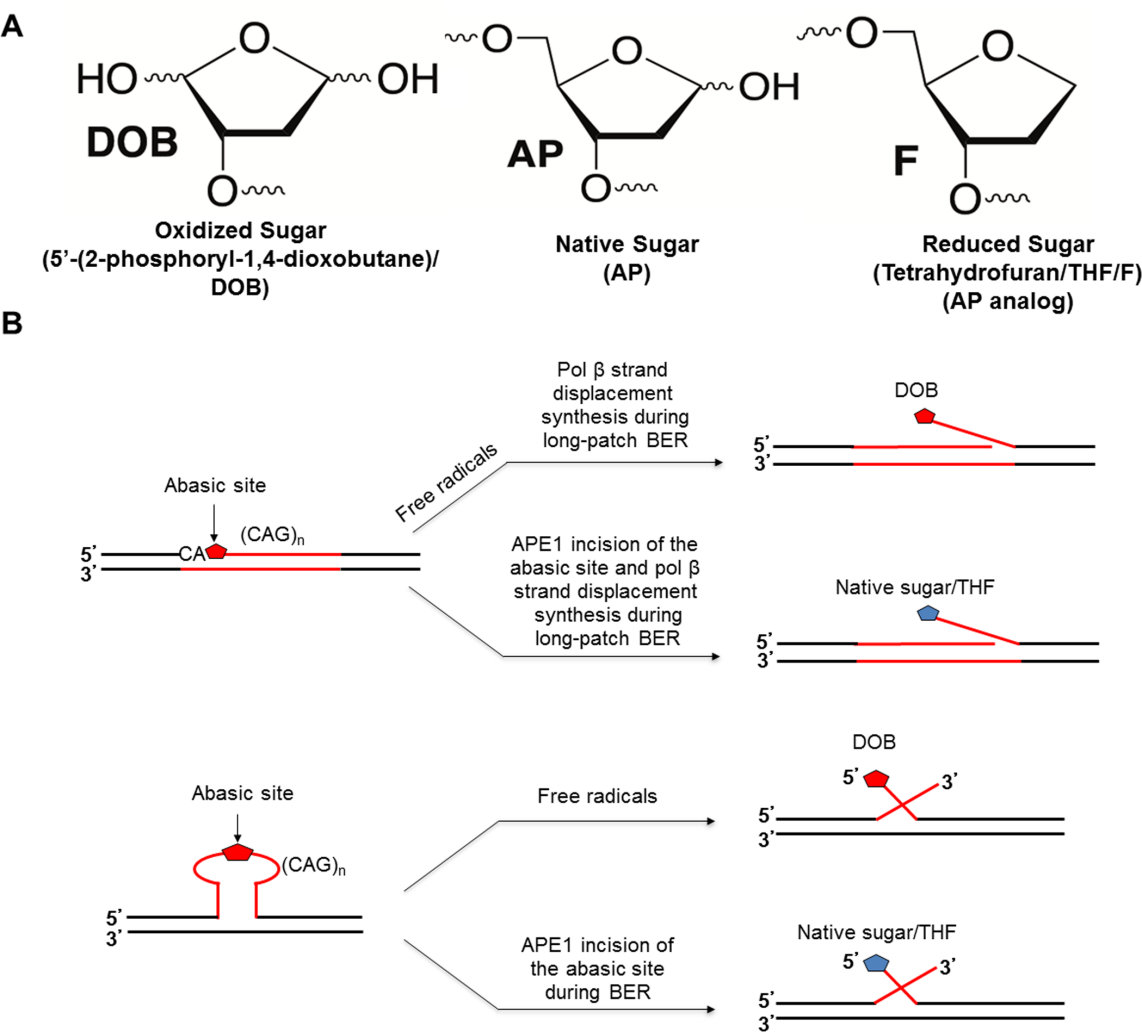
Generation of DNA substrates containing various types of abasic lesions. (A) Abasic DNA lesions with modifications to the sugar residue. An oxidized sugar, DOB (left), native sugar, AP (middle), and reduced sugar, THF (right). (B) Formation of single-flap (above) and double-flap (below) intermediates with various types of sugars during BER.

Oligonucleotide substrates were prepared as described previously [26]. Briefly, substrates mimicking the BER intermediates with a 5’-DOB, 5’-THF, 5’-uracil or 5’-phophate residue were constructed by annealing an upstream primer containing a 3’-(CAG)_3_ flap or a 3’-primer with no repeats and a downstream primer containing a 5’-(CAG)_2_ flap with the template strand containing (CTG)_5_ or (CTG)_2_, respectively, at a molar ratio of 1:3:3. Schematic representations of how these substrates are generated during BER are shown in Fig 1B. Substrates mimicking a BER intermediate containing a nick with a 3’-OH and 5’-phosphate group were constructed by annealing an upstream 15 nt primer with a downstream 16 nt primer and 31 nt template at a ratio of 1:3:3. Oligonucleotide sequences are listed in S1 Table. The uracil-containing substrates were incubated with UDG to create an AP site. The DOB-containing substrates with *o*-nitrobenzyl protecting groups are indicated in S1 Fig [31]. The DOB lesion was generated through photolysis (S1 Fig [31]) by exposing the annealed substrate to 365 nm UV for 20 minutes immediately prior to each experiment. Substrates were labeled with ^32^P at the 5’-end of the upstream strand or 3’-end of the downstream strand as indicated.

### Protein expression and purification

Recombinant human FEN1 was purified as described previously [27]. Briefly, FEN1 was expressed in *E*. *Coli* BL21(DE3). Two one-liter flasks containing lysogeny broth (LB) medium were inoculated with one colony each of the transformed BL21(DE3) cells and were then incubated overnight without shaking. The cultures were then incubated at 37°C at 225 rpm until OD_600_ reached 0.6, and protein expression was induced with 1 mM IPTG for 3.5 hours. Bacterial cells were harvested by centrifugation at 2600 rpm for 45 minutes at 4°C. Cells were lysed with a French press cell disruptor (Glen Mills, Clifton, NJ, USA) at 150,000 PSI in lysis buffer containing 30 mM 4-(2-hydroxyethyl)-1-piperazineethane-sulfonic acid (HEPES), pH 7.5, 30 mM KCl, 1 mM dithiothreitol (DTT), 1 mM EDTA, 1 mM phenylmethylsulfonyl fluoride (PMSF), and 0.5% inositol. The cell lysates were centrifuged at 12,000 rpm for 30 minutes at 4 °C. The supernatant was loaded onto a 10-ml Sepharose Q column operated by an AKTA Fast Protein Liquid Chromatography system (FPLC) (GE Healthcare, Piscataway, NJ, USA). The flow-through was collected and dialyzed into buffer containing 30 mM HEPES, pH 7.5, 30 mM KCl, 0.5% inositol, and 1 mM PMSF, and subsequently loaded onto a 5-ml CM sepharose column (Bio-Rad Laboratories, Hercules, CA, USA), with fractions eluted using a linear gradient of KCl from 30 mM to 2 M. Peak fractions were combined and dialyzed into buffer containing 30 mM HEPES, pH 7.5, 0.5% inositol, 1.7 M (NH_4_)_2_SO_4_, and 1 mM PMSF. Samples were then loaded onto a 2 ml phenyl sepharose column. Fractions were eluted using a linear gradient of (NH_4_)_2_SO_4_ from 1.7 M to 0 M. The peak fractions were combined and dialyzed in buffer containing 30 mM HEPES, pH 7.5, 30 mM KCl, 0.5% inositol, and 1 mM PMSF. Samples were then loaded onto a 1 ml Mono-S column (GE Healthcare, Piscataway, NJ, USA), and eluted using a linear gradient of KCl (30 mM to 2 M). Purified FEN1 was aliquoted and frozen at -80 °C until further use.

Recombinant human LIG I was expressed in *E*. *Coli* BL21(AI). Six one-liter flasks containing LB medium were inoculated with one colony each of the transformed BL21(AI) cells and incubated overnight without shaking. The cells were then incubated at 37°C at 225 rpm until OD_595_ reached 0.6, and LIG I expression was induced by 1 mM IPTG for 24 hours at 15–18 °C. Cells were then harvested by centrifugation at 2500 rpm for 30 minutes at 4 °C. The supernatant was discarded, and cell pellets were lysed in lysis buffer containing 50 mM Tris-HCl, pH 7.5, 50 mM NaCl, 1 mM EDTA, 1 mM DTT, 0.1% Nonidet P-40, 1 mM PMSF, and 1 cOmplete tablet of protease inhibitors (Roche, Indianapolis, IN) and subjected to French Press at 150,000 PSI. The cell lysates were centrifuged at 12,000 rpm for 30 minutes at 4 °C. The supernatant was then loaded onto a 20 ml P11 phosphocellulose column. Fractions were eluted using a linear gradient of NaCl (50 mM to 600 mM). The peak fractions were combined and dialyzed in buffer containing 30 mM HEPES, pH 7.0, 30 mM KCl, 0.1% inositol, and 1 mM PMSF. Samples were loaded onto a 10 ml Q sepharose column and eluted using a linear gradient of KCl (30 mM to 2 M). Peak fractions were combined and dialyzed in buffer containing 50 mM Tris-HCl, pH 8.0, 500 mM NaCl, 7 mM 2-mercaptoethanol, 10 mM imidazole, 0.5% inositol, and 1 mM PMSF. Samples were then loaded onto a 4-ml nickel-nitrilotriacetic acid (Ni-NTA) column, with fractions eluted using a linear gradient of imidazole (10 mM to 600 mM). Purified LIG I was aliquoted and frozen at -80 °C until further use.

### Reconstituted BER assay

*In vitro* BER of the DOB lesion, AP, and the abasic site analog, THF (Fig 1A) at the 5’-end of the downstream primer of the substrates was carried out by incubating 50 nM substrate with the indicated concentrations of FEN1, LIG I, and pol β. AP was generated by pre-incubating the uracil-containing substrate with UDG for 30 minutes. The DOB substrate was produced by photolysis at 365 nm UV light for 20 minutes to remove the protecting groups from the precursor immediately prior to carrying out the reaction. All reactions were carried out in reaction buffer containing 30 mM HEPES, pH 7.8, 50 mM KCl, 0.5% inositol, and 0.1 mg/ml bovine serum albumin (BSA), with 5 mM MgCl_2_, 2 mM ATP, and 50 μM dNTPs. The 20 μl BER reaction mixtures were incubated at 37 °C for 15 minutes, and reactions were terminated by the addition of 20 μl stopping buffer containing 95% formamide and 10 mM EDTA. Reaction mixtures were then denatured at 95 °C for 10 minutes and separated by 15% urea-denaturing polyacrylamide gel electrophoresis. Substrates and products were detected and analyzed using a Pharos FX Plus PhosphorImager from Bio-Rad Laboratories (Hercules, CA, USA). All reactions were done in triplicate.

### BER enzymatic activity assays

The activities of pol β DNA synthesis and FEN1 flap cleavage on all substrates were measured by incubating 50 nM substrates with the indicated concentrations of pol β and FEN1 in reaction buffer containing 30 mM HEPES, pH 7.8, 50 mM KCl, 0.5% (w/v) inositol, and 0.1 mg/ml BSA, with 5 mM MgCl_2_ and 50 μM dNTPs in a total volume of 20 μl. For the pol β DNA synthesis assay, the 20 μl BER reaction mixtures were incubated at 37 °C. Aliquots were removed at time points ranging from 2–30 minutes, and the reactions were quenched by the addition of 20 μl of 2 x stopping buffer containing 95% formamide and 10 mM EDTA. For the FEN1 5’-flap cleavage activity assay, samples were preincubated with 2.5 nM pol β for 1–5 minutes to allow for crosslink formation prior to incubation with FEN1 for 15 minutes at 37 °C. Reactions were quenched with 200 nM NaBH_4_ on ice for 30 minutes. Reaction mixtures were then denatured at 95 °C for 10 minutes and separated by 15% urea-denaturing polyacrylamide gel electrophoresis. Substrates and products were detected and analyzed using a Pharos FX Plus PhosphorImager.

LIG I activity was tested on a substrate of random DNA sequence containing a central nick with a phosphate at the 5’-end of the downstream strand in reaction buffer containing 30 mM HEPES, pH 7.8, 50 mM KCl, 0.5% inositol, and 0.1 mg/ml BSA, with 5 mM MgCl_2_ and 4 mM ATP. A single strand containing either a 5’-DOB or 5’-THF was preincubated with LIG I for 30 minutes prior to addition of the nicked ligatable substrate. Reaction mixtures (20 μl) were incubated for 15 minutes at 37 °C. Reactions were quenched by the addition of 20 μl of 2 x buffer containing 95% formamide and 10 mM EDTA. Reaction mixtures were then denatured at 95 °C for 10 minutes and separated by 15% urea-denaturing polyacrylamide gel electrophoresis. Substrates and products were detected and analyzed using a Pharos FX Plus PhosphorImager.

### Detection of BER enzyme-DNA crosslink using a NaBH_4_ trapping assay

The pol β-DNA crosslink was captured by incubating 1 μM of pol β wild-type or K72A mutant protein with 2.5 nM DOB- or AP-containing nicked 5’-(CAG)_2_ flap substrates and (CAG)_3_/(CAG)_2_ double-flap substrates in reaction buffer containing 30 mM HEPES, pH 7.8, 50 mM KCl, 0.5% inositol, 0.1 mg/ml BSA, 10 mM MgCl_2_ and 1 mM 2-mercaptoethanol in the absence and presence of 100 mM NaBH_4_. The LIG I-DOB crosslink was captured by incubating 1 μM of LIG I with 1 μM 5’-DOB containing single strand CAG repeat DNA in reaction buffer containing 30 mM HEPES, pH 7.8, 50 mM KCl, 0.5% inositol, 0.1 mg/ml BSA, 10 mM MgCl_2_ and 1 mM 2-mercaptoethanol in the absence and presence of 20, 50, or 100 mM NaBH_4_. The native sugar was generated by pre-incubating the uracil-containing substrate with UDG for 30 minutes. The photolyzed DOB-containing substrates were subjected to photolysis at 365 nm UV light for 20 minutes to remove the protecting groups for creating a DOB lesion immediately prior to the crosslink reaction. Reaction mixtures (20 μl) were incubated for 1 hr at 37 °C, and the reactions were terminated by adding an equal volume of SDS loading buffer containing 100 mM Tris-HCl, pH 6.8, 4% (w/v) SDS, 20% (v/v) glycerol, 0.2% (w/v) bromophenol blue, and 200 mM dithiothreitol. The samples were then heated at 95 °C for 3 min, and crosslink products were separated by 10% SDS-PAGE. Substrates and products were detected and analyzed using a Pharos FX Plus PhosphorImager.

### Statistical analysis

Statistical analysis was performed using GraphPad Prism 6 (Graphpad software, San Diego, CA). Significant differences in the data were examined by standard two-way analysis of variance with Tukey’s multiple comparison posttests. The significant difference was designated at *P* < 0.05.

## Results

### The oxidized abasic lesion, DOB inhibits pol β synthesis activity during BER in a CAG repeat tract

The DOB lesion residing in the 1-nt gap of duplex DNA forms a covalent crosslink with the deoxyribosephosphate (dRP) lyase domain of pol β, resulting in irreversible enzyme inactivation [28]. However, it remains unknown if inhibition of pol β by DOB-pol β crosslink exhibits an adverse impact on DNA repair and genome stability, such as repeat sequence instability. To address this, we asked whether this crosslink affects BER efficiency and TNR instability when the DOB lesion is present in a TNR tract. The repeat tract can form various secondary structures due to DNA slippage, which may position the DOB lesion in proximity to BER enzymes. This would facilitate DOB crosslinking with pol β and thus affect pol β synthesis activity, BER efficiency, and TNR instability during BER in a TNR tract. To test this possibility, we initially measured pol β DNA synthesis activity on CAG repeat substrates containing DOB and compared this to substrates containing a native sugar (AP) or THF at the 5’-end of the downstream flap (Fig 1A). These substrates mimic the intermediates generated during BER of an abasic lesion in a duplex TNR tract or a TNR hairpin in cells (Fig 1B). Fig 1 illustrates a scheme showing the formation of the intermediates containing a DOB lesion, AP and reduced sugar (THF) during BER in a duplex TNR tract and TNR hairpin in cells. Cellular exposure to ionizing radiation or potent antitumor antibiotics such as neocarzinostatin, produce free radicals that abstract a hydrogen atom from the C5' position of the deoxyribose, generating a single-strand break and a DOB lesion located at the 5’-end of the break [32–35]. Since the DOB lesion is an oxidized sugar, which is refractory to pol β dRP lyase activity due to the fact that only a native sugar, dRP [36] can be directly removed by the dRP lyase activity via β-elimination [37] during short-patch BER, the lesion can only be removed through the long-patch BER subpathway, during which pol β performs multi-nucleotide gap-filling synthesis and/or strand displacement synthesis and coordinates with FEN1 removal of a 5’-flap that contains 5’-DOB. A substrate containing a nick with a downstream (CAG)_2_-flap (Fig 1B) was used to mimic the intermediate generated by pol β strand-displacement synthesis during BER in a CAG repeat duplex. A (CAG)_3_/(CAG)_2_ double-flap substrate (Fig 1B) was used to mimic the intermediate formed following APE1 5'-incision of an abasic site in a (CAG)_5_ hairpin. Pol β DNA synthesis activity on these substrates was tested at a time interval ranging from 2–30 minutes.

**Fig 2:**
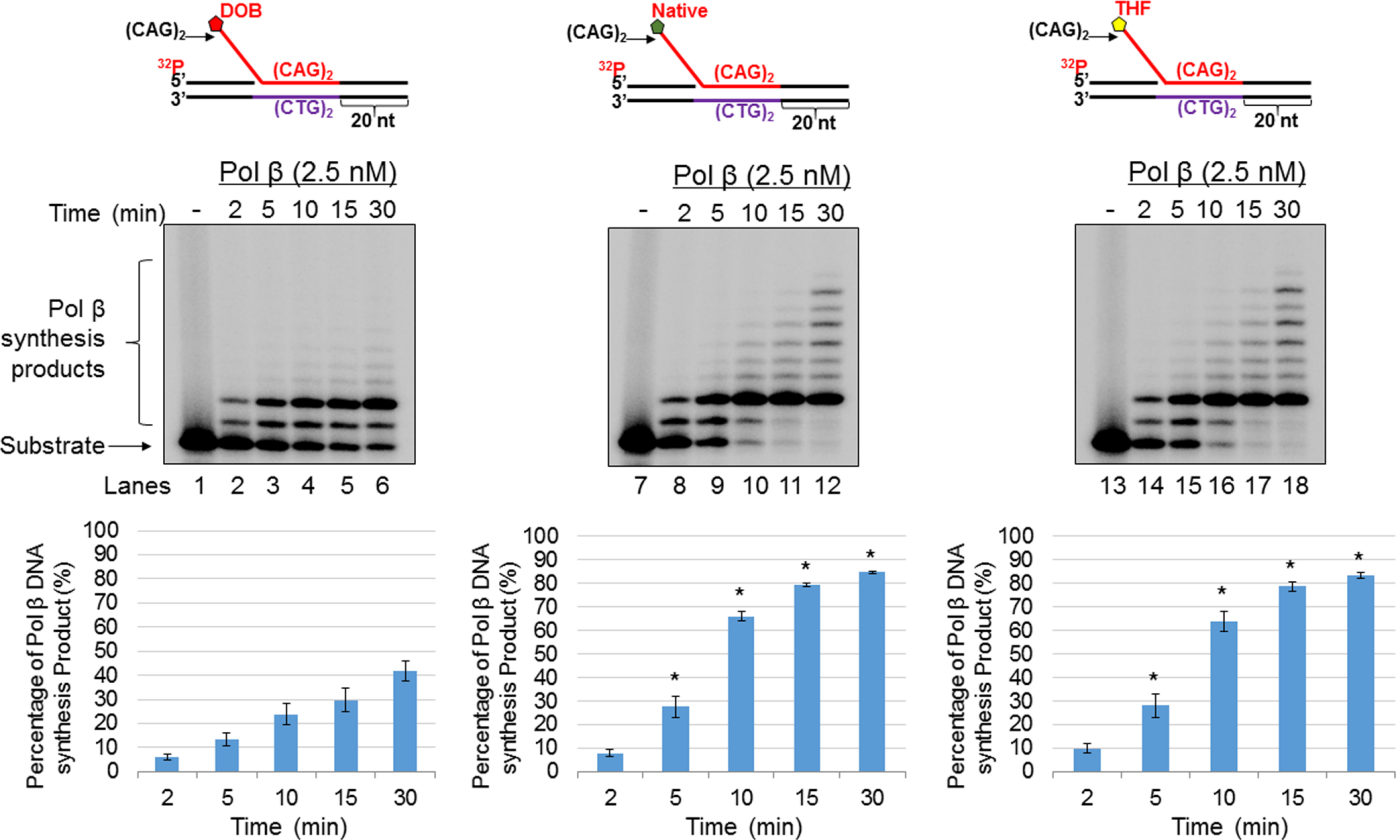
The oxidized abasic lesion DOB inhibits pol β DNA synthesis activity during BER in a CAG repeat duplex. The effect of the type of abasic lesion on pol β synthesis activity with a substrate containing a single (CAG)_2_ downstream flap formed during BER of a CAG repeat duplex was examined by measuring pol β synthesis activity on the substrate containing a DOB (left panel), native sugar (middle panel), or THF (right panel). Lanes 1, 7, and 13 indicate the substrate only. Lanes 2–6, lanes 8–12, and lanes 14–18 indicate the substrate incubated with pol β (2.5 nM) for a time course of 2, 5, 10, 15, and 30 minutes. Substrates were ^32^P-labeled at the 5’-end of the upstream strand and are illustrated above each gel. The experiments were repeated at least three times. Representative gels are shown. The quantification of the data is presented below the gels. Two-way ANOVA with Tukey’s multiple comparison posttests was used to determine statistically significant differences. "*" denotes *P* < 0.05, compared to the DOB-containing substrates.

**Fig 3:**
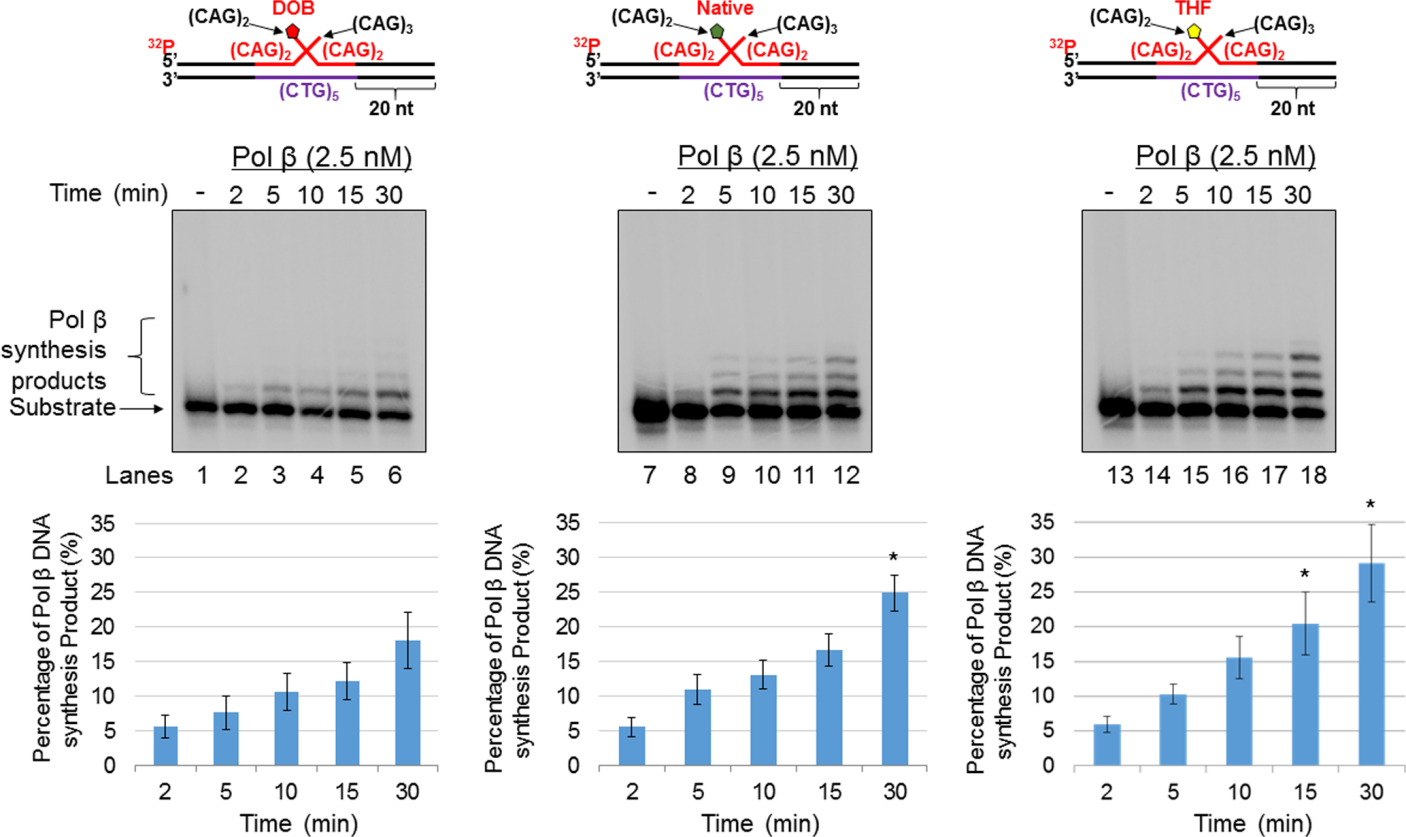
The oxidized abasic lesion DOB inhibits pol β DNA synthesis activity during BER in a small CAG hairpin loop. The effect of the type of abasic lesion on pol β synthesis activity with a short (CAG)_3_/(CAG)_2_ double-flap intermediate formed during BER of a (CAG)_5_ hairpin was examined by measuring pol β synthesis activity on the substrate containing a DOB (left panel), native sugar (middle panel), or THF (right panel). Lanes 1, 7, and 13 indicate the substrate only. Lanes 2–6, lanes 8–12, and lanes 14–18 indicate the substrate incubated with pol β (2.5 nM) for a time course of 2, 5, 10, 15, and 30 minutes. Substrates were ^32^P-labeled at the 5’-end of the upstream strand and are illustrated above each gel. The experiments were repeated at least three times. Representative gels are shown. The quantification of the data is presented below the gels. Two-way ANOVA with Tukey’s multiple comparison posttests was used to determine statistically significant differences. "*" denotes *P* < 0.05, compared to the DOB-containing substrates.

**Fig 4:**
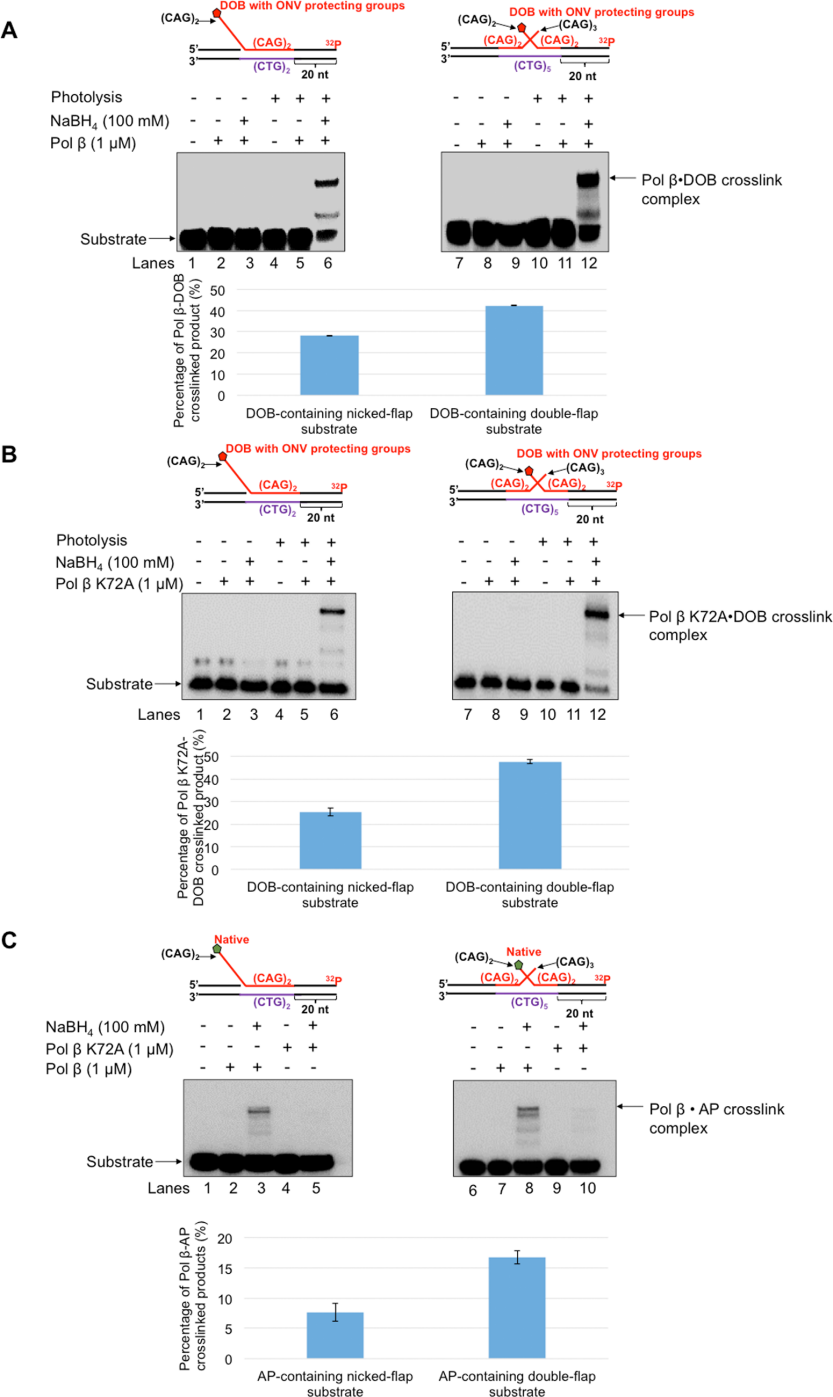
Wild-type pol β crosslinks with DOB and AP, whereas pol β K72A mutant only crosslinks with DOB. The formation of a pol β•DOB crosslink complex (**A)** and pol β K72A mutant•DOB crosslink complex (**B)** was measured using the nicked flap substrates and double-flap substrates containing DOB as described in the “Methods”. Lanes 1 and 7 contain the unphotolyzed substrate only. Lanes 2 and 8 represent reaction mixtures with the unphotolyzed substrates and pol β (1 μM). Lanes 3 and 9 indicate reaction mixtures containing the unphotolyzed substrates and pol β (1 μM) in the presence of 100 mM NaBH4. Lanes 4 and 10 indicate the photolyzed substrate only. Lanes 5 and 11 indicate reaction mixtures with the photolyzed substrates and pol β (1 μM). Lanes 6 and 12 indicate reaction mixtures containing the photolyzed substrates and pol β (1 μM) in the presence of 100 mM NaBH_4_. (**C**) The formation of pol β•AP crosslink and pol β K72A mutant•AP crosslink was examined using the nicked flap substrates and double-flap substrates containing a 5’-uracil as described in the “Methods”. Lanes 1 and 6 indicate the substrate only. Lanes 2 and 7 indicate reaction mixtures with 1 μM pol β only. Lanes 3 and 8 indicate reaction mixtures containing 1 μM pol β in the presence of 100 mM NaBH_4_. Lanes 4 and 9 indicate reaction mixtures with 1 μM pol β K72A mutant only. Lanes 5 and 10 indicate reaction mixtures with 1 μM pol β K72A mutant in the presence of 100 mM NaBH_4_. Substrates were ^32^P-labeled at the 3’-end of the downstream strand and are illustrated above each gel. Each experiment was done in triplicate.

For the nicked-flap substrate with a DOB, 2.5 nM pol β mainly inserted up to 2 nucleotides (Fig 2, lanes 2–6). Although the same concentration of pol β mainly inserted 2 nucleotides when acting on nicked-flap substrates containing AP or THF, some of the synthesis products were further extended up to 8 nt (Fig 2, lanes 8–12 and lanes 14–18). The quantitative results showed that the pol β synthesis products generated from the substrates containing AP or THF, were increased 2-3-fold compared with the products generated from the substrate containing a DOB (Fig 2, the bar charts below the gels) (*P*<0.05). For the double-flap substrate, pol β mainly inserted 1 nucleotide on the DOB substrate (Fig 3, lanes 2–6), whereas it inserted up to 3 nucleotides on the substrates containing AP or THF (Fig 3, lanes 8–12 and lanes 14–18). An increase in the pol β synthesis products from the substrates containing AP or THF compared with the DOB-containing substrate was detected at 15 and 30 min (Fig 3, the bar charts below the gels). In addition, pol β exhibited similar DNA synthesis on the substrates with a 5'-phosphate at the CAG repeat flaps to that on the substrates containing AP or THF (S2 Fig). The results consistently indicated that a DOB lesion on a CAG repeat flap inhibited pol β DNA synthesis activity during BER. Based upon what is known about DOB reactivity, we speculated that the lesion located at the end of a 5’-flap crosslinked with pol β, even though the lesion was away from the gap on the duplex DNA [29]. We employed a borohydride trapping assay to determine if a crosslink formed between pol β and a DOB lesion on the 5'-end of the CAG repeat flaps. We detected a pol β-DOB crosslink on the nicked-flap and double-flap substrates (Fig 4A, lanes 6 and 12). Crosslink formation was detected only when the DOB-containing substrates, pol β and borohydride (NaBH_4_) were present simultaneously (Fig 4A, lanes 6 and 12). Crosslinking was not observed in the absence of a DOB lesion, in the presence of DOB precursor, or under various other conditions lacking one or more reactants, including NaBH_4_ (Fig 4A). To determine if the DOB lesion at a CAG repeat flap specifically crosslinked with lysine 72 (K72) of pol β as it does on a 1 nt-gapped substrate [29], we examined the crosslink between the pol β K72A mutant protein and a DOB lesion. Surprisingly, we found that the mutant pol β formed a crosslink complex with the DOB lesion in similar amounts compared with wild-type pol β (Fig 4B, lanes 6 and 12), indicating that DOB crosslinked with other lysines in the catalytic site of the pol β dRP lyase domain. Similar to the wild-type pol β, the pol β K72A mutant failed to form a crosslink complex with the substrates under conditions in which any of the reaction components were missing (Fig 4B, lanes 2–3, lane 5, lanes 8–9 and lane 11). In contrast, the native sugar (AP) crosslinked with wild-type pol β protein (Fig 4C, lanes 3 and 8) but failed to crosslink with the pol βK72A mutant protein (Fig 4C, lanes 5 and 10). This suggests that the transient crosslink between AP and pol β was solely mediated by K72. Moreover, we found that pol β that was crosslinked with DOB, lost its dRP lyase activity (S3 Fig, lanes 7 and 9 and quantitative data), whereas uncrosslinked pol β retained efficient dRP lyase activity (S3 Fig, lanes 5, 6 and 8 and quantitative data). The data consistently indicate that the DOB lesion at a CAG repeat flap crosslinked with Lys72 and/or other lysines in pol β, thereby inhibiting its DNA synthesis and dRP lyase activities during BER in CAG repeat tracts. In contrast, pol β was only transiently crosslinked at K72 by the native AP site. It should be noted that with a relatively high concentration of pol β, we successfully detected the DOB-pol β crosslink product in a SDS-PAGE gel. This suggests that a high level of pol β proteins was required to generate a sufficient amount of pol β-DOB crosslink products to be detected by SDS-PAGE. It should be also noted that in the results, the multiple crosslink species in the gels were observed. This may be due to the formation of a series of secondary structures by the CAG repeats that migrated differently in a SDS-PAGE gel.

### The oxidized abasic lesion, DOB did not significantly affect FEN1 cleavage of CAG repeats during BER

**Fig 5:**
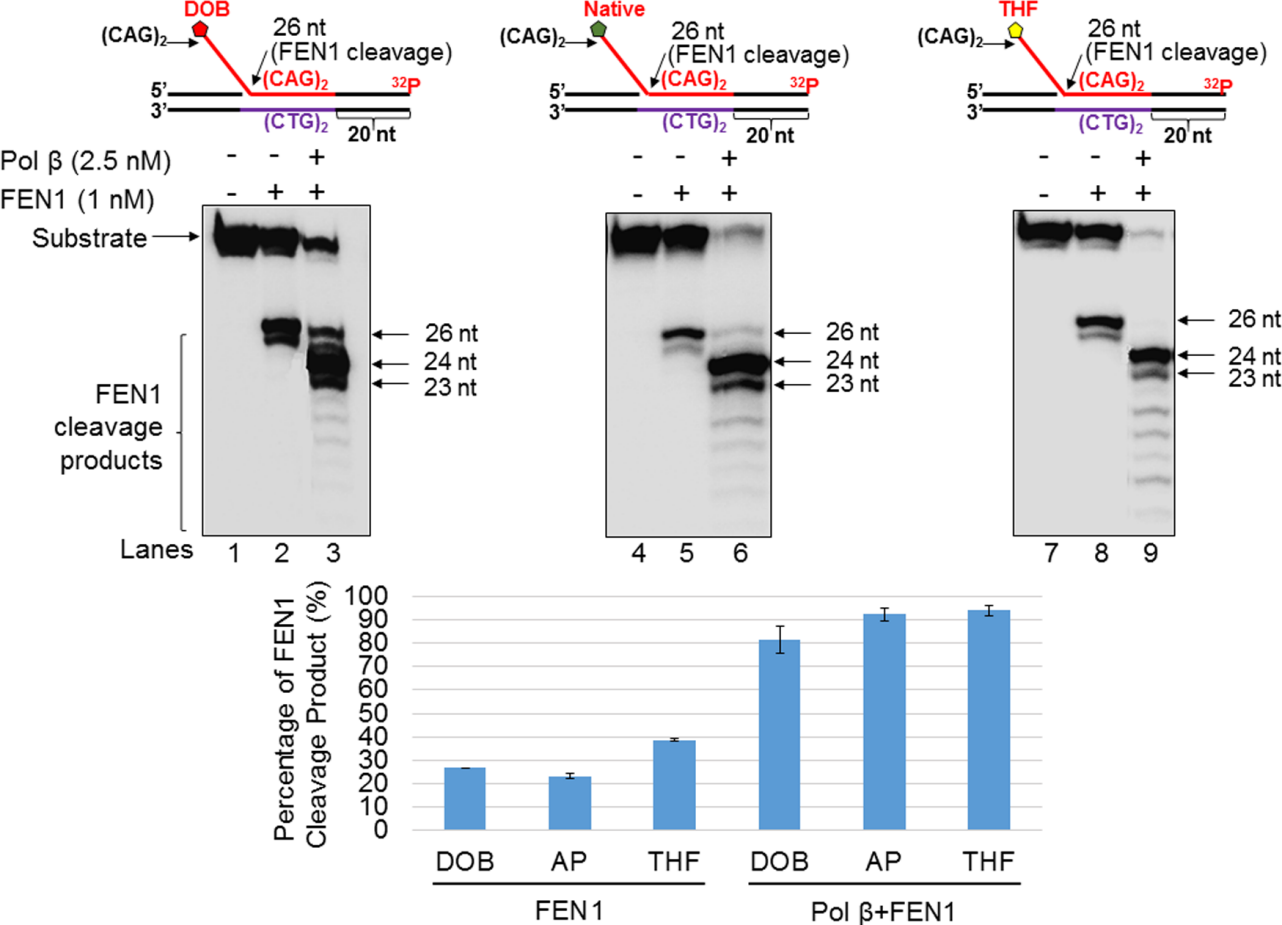
The oxidized abasic lesion does not significantly affect FEN1 5’-flap cleavage activity during BER in a CAG repeat duplex. The effect of the pol β-DOB crosslink on FEN1 cleavage activity on a substrate containing a single (CAG)_2_ downstream flap intermediate formed during BER of CAG repeat duplex was explored by measuring FEN1 cleavage activity on the substrate containing a DOB (left panel), native sugar (middle panel), or THF (right panel). Lanes 1, 5, and 9 indicate substrate only. Lanes 2, 6, and 10 indicate substrate incubated with FEN1 (1 nM) only. Lanes 3, 7, and 8 indicate the substrate pre-incubated with pol β (2.5 nM) for 1 min prior to incubation with FEN1 (1 nM). Lanes 4, 8, and 12 indicate substrates pre-incubated with pol β (2.5 nM) for 5 min prior to incubation with FEN1 (1 nM). Substrates were ^32^P-labeled at the 3’-end of the upstream strand and are illustrated above each gel. The experiments were repeated at least three times. Representative gels are shown. The quantification of the data is presented below the gels. Two-way ANOVA with Tukey’s multiple comparison posttests was used to determine statistically significant differences. "*" denotes P < 0.05, compared to the DOB-containing substrates.

**Fig 6:**
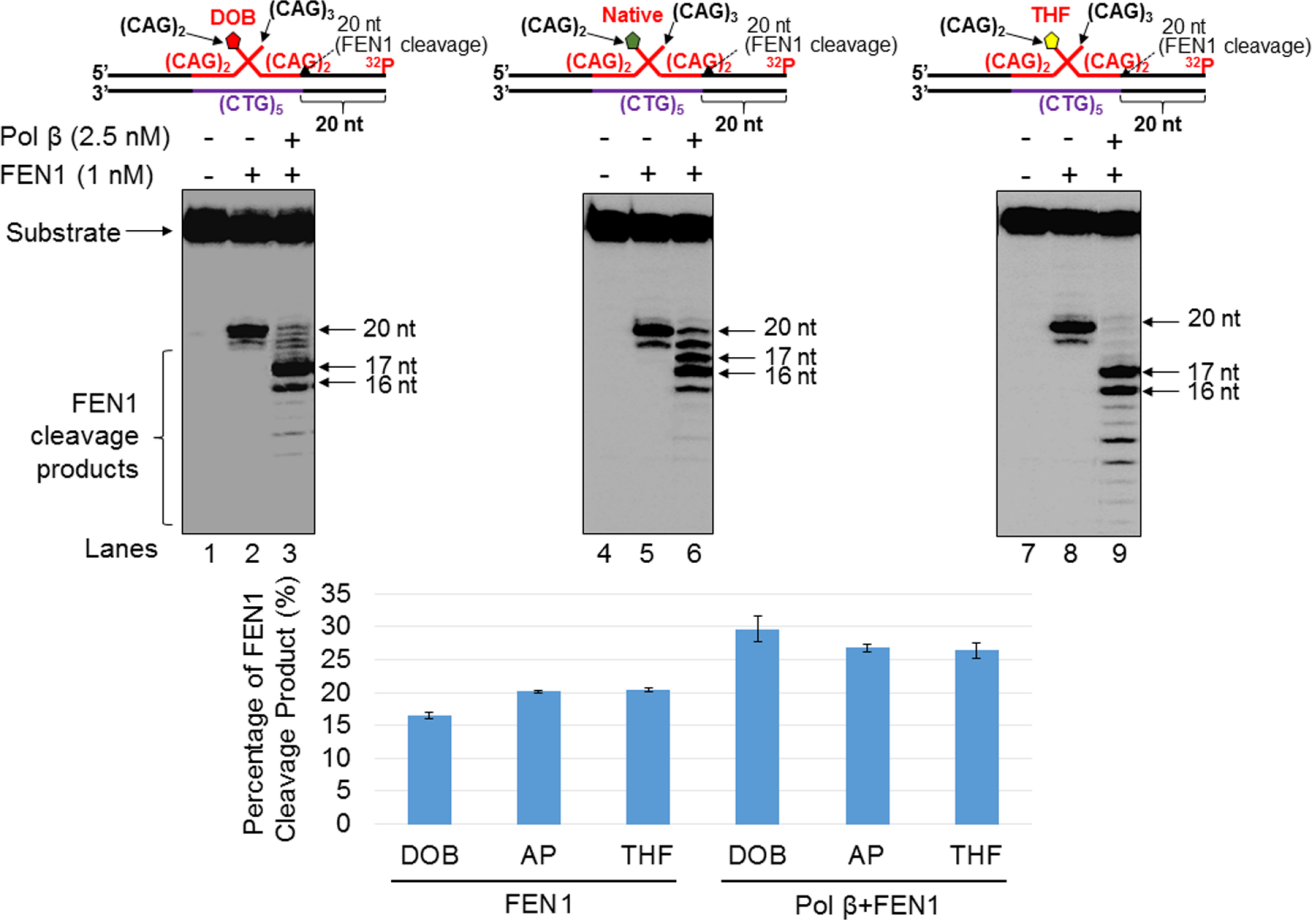
The oxidized abasic lesion does not affect FEN1 5’-flap cleavage activity during BER in a small CAG hairpin loop. The effect of the pol β-DOB crosslink on FEN1 cleavage activity on a short (CAG)_3_/(CAG)_2_ double-flap intermediate formed during BER of a (CAG)_5_ hairpin was examined by measuring FEN1 cleavage activity on the substrate containing a DOB (left panel), native sugar (middle panel), or THF (right panel). Lanes 1, 5, and 9 indicate the substrate only. Lanes 2, 6, and 10 indicate substrates incubated with FEN1 (1 nM) only. Lanes 3, 7, and 8 indicate substrates pre-incubated with pol β (5 nM) for 1 min prior to incubation with FEN1 (1 nM). Lanes 4, 8, and 12 indicate substrates pre-incubated with pol β (5 nM) for 5 min prior to incubation with FEN1 (1 nM). Substrates were ^32^P-labeled at the 3’-end of the upstream strand and are illustrated above each gel. The experiments were repeated at least three times. Representative gels are shown. The quantification of the data is presented below the gels. Two-way ANOVA with Tukey’s multiple comparison posttests was used to determine statistically significant differences. "*" denotes P < 0.05, compared to the DOB-containing substrates.

We then sought to determine whether DOB inhibition of pol β mediated DNA synthesis affects FEN1 5’-flap cleavage. We therefore tested FEN1 cleavage activity on the substrate containing a nick with a downstream (CAG)_2_-flap (Fig 5) formed during BER in a duplex TNR tract, as well as on the (CAG)_3_/(CAG)_2_ double-flap substrate (Fig 6), which mimics the intermediate generated after APE1 5'-incision of an abasic site in a (CAG)_5_ hairpin. For the nicked-flap substrate, in the absence of pol β, FEN1 cleavage on the substrates with different types of sugar lesions resulted in a similar amount of the 26 nt product (23.1%-38.4%) (Fig 5, lanes 2, 5 and 8 and the bar charts below the gels). In the presence of pol β, FEN1 cleavage on the nicked substrate mainly resulted in products that are ≤ 24 nt (81.8% from the DOB substrate, 93.3% from the native sugar substrate and 94.4% from the THF substrate) (Fig 5, lanes 3, 6 and 9). For the double-flap substrate, FEN1 alone resulted in a 20 nt product (16.4% for the DOB substrate, 20.1% for the native sugar substrates and 20.5% for the THF substrate) (Fig 6, lanes 2, 5, and 8). In the presence of pol β, FEN1 cleavage on the substrates generated ≤ 20 nt products (29.6% for the DOB substrate, 26.8% for the native sugar substrates and 26.4% for the THF substrate) (Fig 6, lanes 3, 6, and 9). The results indicate that DOB did not significantly affect FEN1 cleavage of CAG repeats during BER.

### The oxidized abasic lesion, DOB reduces BER efficiency in a CAG repeat tract

**Fig 7:**
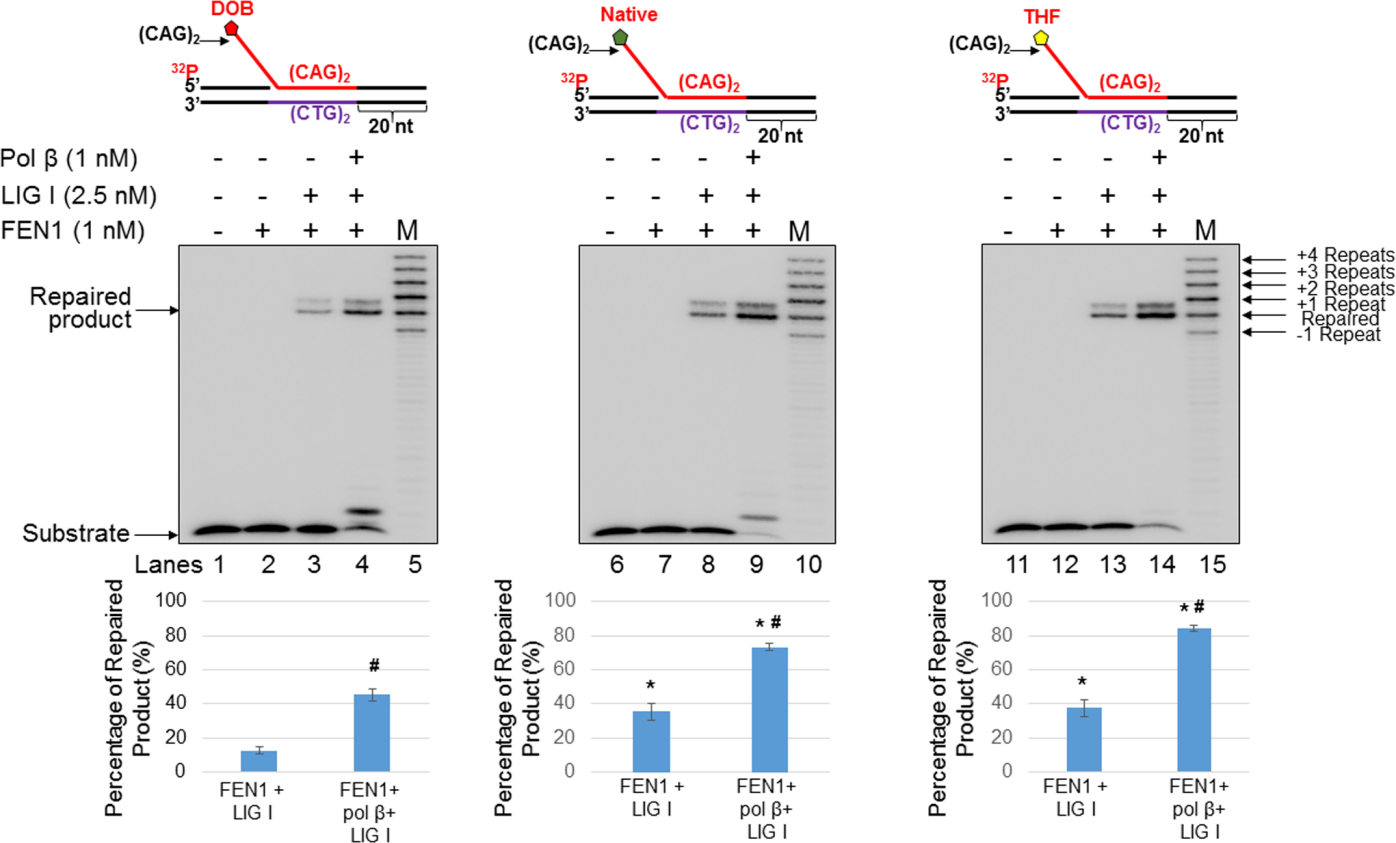
An oxidized abasic lesion (DOB) decreases BER efficiency in a CAG repeat duplex. The effect of abasic lesion type on BER efficiency during BER in a CAG repeat duplex was examined by reconstituting BER with a substrate containing a single (CAG)_2_ downstream flap intermediate formed during BER of a CAG repeat duplex containing a DOB (left panel), native sugar (middle panel), or THF (right panel). Lanes 1, 6, and 11 indicate the substrate only. Lanes 2, 7, and 12 indicate the substrate incubated with FEN1 (1 nM) only. Lanes 3, 8, and 13 indicate reaction mixtures incubated with FEN1 (1 nM) and LIG I (2.5 nM). Lanes 4, 9, and 14 indicate reaction mixtures incubated with FEN1 (1 nM), LIG I (2.5 nM), and pol β (1 nM). Lanes 5, 10, and 15 indicate synthesized size markers as indicated to the left of the gel. Graphs indicating the percentage of repaired products are shown below each gel. Each experiment was done in triplicate, and only the representative gels are shown in the figures. Substrates were ^32^P-labeled at the 5’-end of the upstream strand and are illustrated above each gel. Percentage of repair product (%) = intensity of repaired product/(intensity of repaired product + intensity of residual substrate)×100. The quantification data are illustrated below the gels. Two-way ANOVA with Tukey’s multiple comparison posttests was used to determine statistically significant differences. "*" denotes *P* < 0.05, compared to DOB-containing substrates. "#" denotes *P* < 0.05, compared to repair reactions in the absence of pol β.

**Fig 8:**
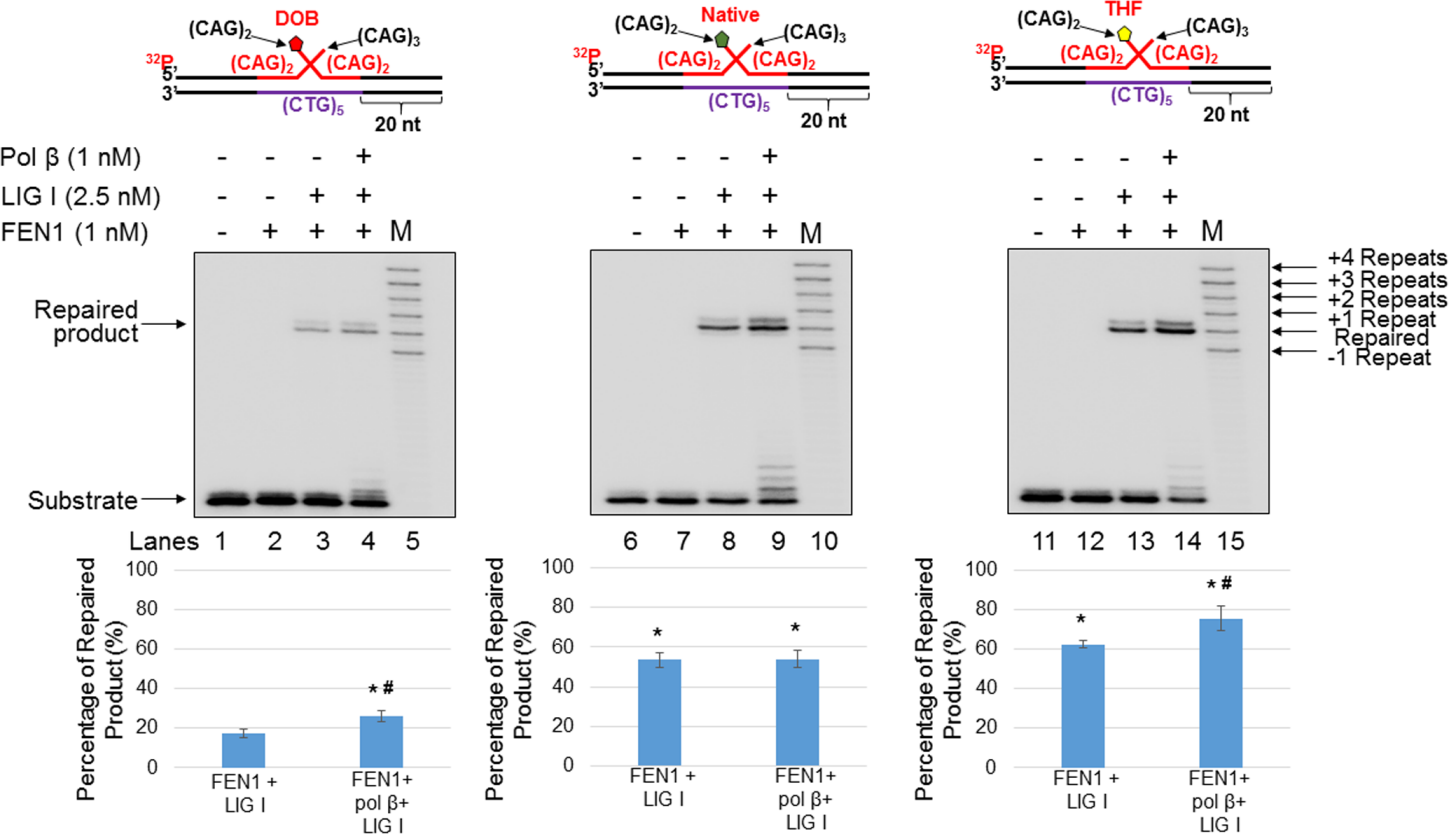
An oxidized abasic lesion (DOB) decreases repair efficiency during BER in a small CAG hairpin loop. The effect of abasic lesion type on BER efficiency during BER in a small CAG hairpin loop was examined by reconstituting BER with the short (CAG)_3_/(CAG)_2_ double-flap intermediate formed during BER in a (CAG)_5_ hairpin containing a DOB (left panel), native sugar (middle panel), or THF (right panel). Lanes 1, 6, and 11 indicate the substrate only. Lanes 2, 7, and 12 indicate the substrate incubated with FEN1 (1 nM) only. Lanes 3, 8, and 13 indicate reaction mixtures incubated with FEN1 (1 nM) and LIG I (2.5 nM). Lanes 4, 9, and 14 indicate reaction mixtures incubated with FEN1 (1 nM), LIG I (2.5 nM), and pol β (1 nM). Lanes 5, 10, and 15 indicate synthesized size markers as indicated to the left of the gel. Graphs indicating the percentage of repaired products are shown below each gel. Each experiment was done in triplicate, and only the representative gels are shown in the figures. Substrates were ^32^P-labeled at the 5’-end of the upstream strand and are illustrated above each gel. Percentage of repair product (%) = intensity of repaired product/(intensity of repaired product + intensity of residual substrate)×100. The quantification data are illustrated below the gels. Two-way ANOVA with Tukey’s multiple comparison posttests was used to determine statistically significant differences. "*" denotes *P* < 0.05, compared to the DOB-containing substrates. "#" denotes *P* < 0.05, compared to repair reactions in the absence of pol β.

**Fig 9:**
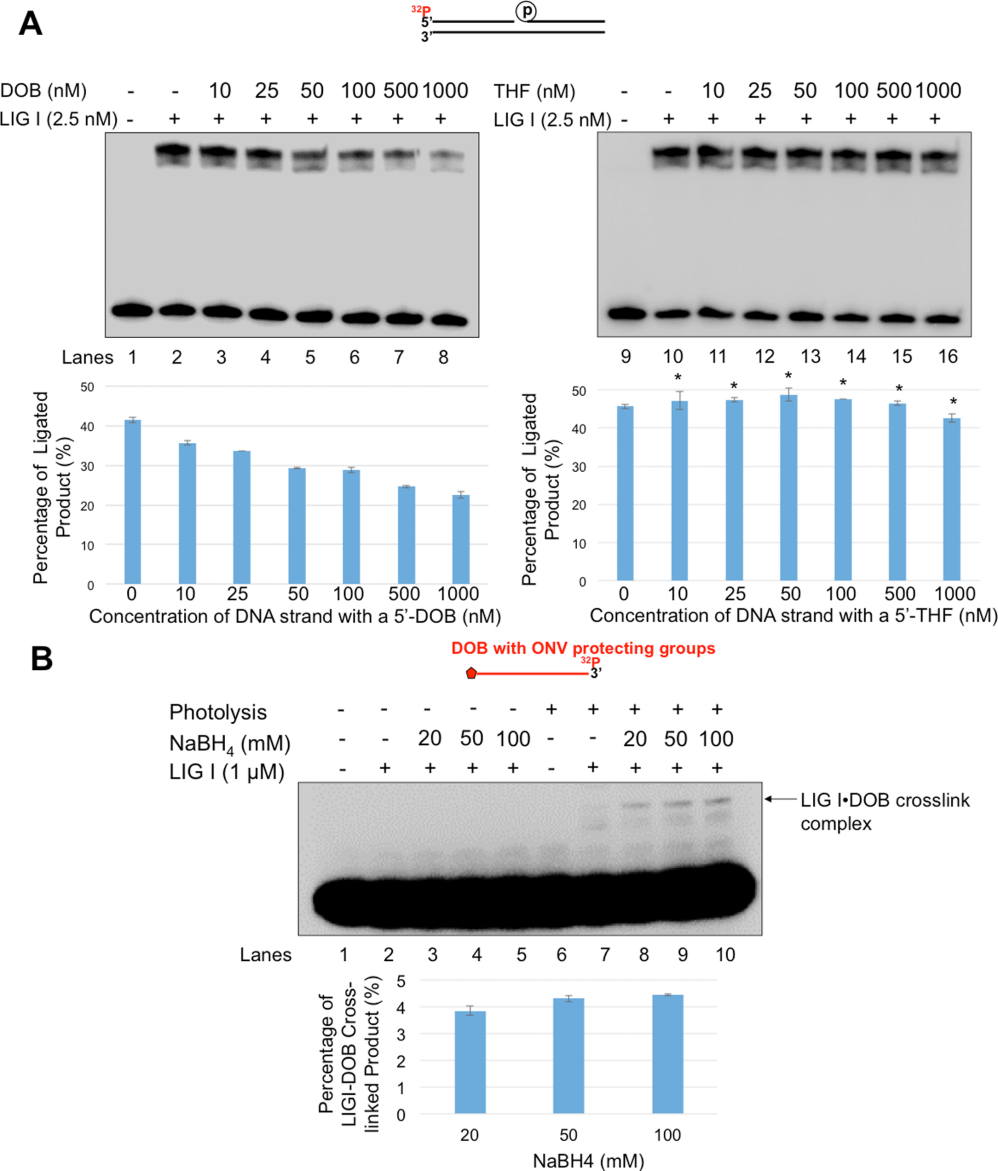
An oxidized abasic lesion inhibits LIG I activity by a DOB-LIG I crosslink. (**A**) The effect of an oxidized lesion on LIG I activity was examined by incubating the nicked DNA substrate with LIG I in the presence of increasing concentrations of DOB—(left panel) or THF—(right panel) containing DNA (0–1000 nM). Lanes 1 and 9 indicate the substrate alone. Lanes 2 and 10 indicate the substrate incubated with 2.5 nM LIG I only. Lanes 3–8 and lanes 11–16 indicate the substrate incubated with 2.5 nM LIG I and increasing concentrations of lesion-containing DNA (0–1000 nM), as indicated. Graphs indicating the percentage of ligated products are shown below each gel. Substrates were ^32^P-labeled at the 5’-end of the upstream strand and are illustrated above each gel. Two-way ANOVA with Tukey’s multiple comparison posttests was used to determine statistically significant differences. "*" denotes *P* < 0.05, compared to the DOB-containing substrates. (**B)** The formation of a LIG I•DOB crosslink complex was determined as described in the “Methods”. Lane 1 indicates the unphotolyzed substrate only. Lane 2 indicates reaction mixtures with the unphotolyzed substrates and LIG I (1 μM). Lanes 3–5 indicate reaction mixtures containing the unphotolyzed substrates and LIG I (1 μM) in the presence of 20, 50, and 100 mM NaBH4, respectively. Lane 6 indicates the photolyzed substrate only. Lane 7 indicates the reaction mixtures with photolyzed substrates and LIG I (1 μM). Lanes 8–10 indicate reaction mixtures containing the photolyzed substrates and LIG I (1 μM) in the presence of 20, 50, and 100 mM NaBH4, respectively. Each experiment was done in triplicate, and only representative gels are shown in the figures.

To further determine whether the reduced pol β activity resulting from an oxidized abasic site affects BER efficiency in a TNR tract, we reconstituted BER with the substrate containing a nick with a downstream (CAG)_2_-flap (Fig 7), as well as with the (CAG)_3_/(CAG)_2_ double-flap substrate (Fig 8), containing a DOB, AP, or THF at the 5’-end of the downstream flap. We found that during BER of a DOB lesion in the substrate containing a nicked-(CAG)_2_-flap (Fig 7), the oxidized abasic site resulted in a significant decrease in the formation of the repaired product (Fig 7, compare lanes 3–4 with lanes 8–9 and lanes 13–14, quantification of the repaired product is shown below each gel) (*P*<0.05). The results indicate that BER efficiency is decreased by the oxidized abasic site, leading to the accumulation of unligated DNA strand break intermediates. For the (CAG)_3_/(CAG)_2_ double-flap substrate (Fig 8), the amount of repaired product from the DOB-containing substrate was reduced ~2-fold compared to that containing AP or THF (Fig 8, compare lanes 3–4 with lanes 8–9 and lanes 13–14, percentage of the repaired product is shown below each gel) (*P*<0.05). This suggests that DOB also inhibited repair product formation with the double-flap substrate, possibly by preventing the formation of a ligatable nick suitable for ligation by LIG I. Our results also showed that DOB significantly decreased the amount of the repaired products in the absence of pol β compared to that from a native sugar or THF lesion (Figs 7 and 8, compare lane 3 with lanes 8 and 13), and more repaired products were generated in the presence of pol β when compared to that in the absence of pol β during BER on the substrates containing a nick with a downstream (CAG)_2_-flap (Fig 7, compare lane 4 and 3, lane 9 and 8, and lane 14 and 13). This indicates that FEN1 cleavage of the downstream flap yielded an intermediate containing a one-nucleotide gap that required pol β gap-filling synthesis to generate a nick for ligation. The decrease in the amount of the repaired product in the absence of pol β (Figs 7 and 8, compare lane 3 with lanes 8 and 13) suggests that the activity of LIG I was also inhibited by the oxidized abasic site, as FEN1 removed the 5’-DOB-containing flap as efficiently as it removes a 5’-AP- or 5’-THF-containing flap in the absence of pol β (Figs 5 and 6, compare lane 2 with lanes 5 and 8). In contrast, little difference in amount of the repaired products for the (CAG)_3_/(CAG)_2_ double-flap substrates was observed (Fig 8, lane 4 and 3, lane 9 and 8, and lane 14 and 13), presumably due to reannealing of the upstream flap to the template strand to yield nicks suitable for ligation in the absence of pol β. It should be noted that our results showed that FEN1 flap cleavage activity was unaffected by DOB *per se* (Figs 5 and 6, compared lane 2 to lanes 5 and 8). However, our results showed that the DOB-containing ssDNA flaps that were cleaved by FEN1 also inhibited the ligation of LIG I (Fig 9). Taken together, the DOB lesion inhibited BER by inhibiting pol β mediated DNA synthesis and ligation by LIG I, thereby reducing the production of repaired (ligated) products.

### The oxidized abasic lesion, DOB, inhibits LIG I activity

The inhibition of the formation of the BER product by DOB suggested that LIG I was also inhibited by the lesion. Since LIG I preferentially binds to a nick, it cannot directly interact with the oxidized sugar at the 5'-end of a flap. However, the enzyme may interact and react with a single strand containing a 5’-DOB after the flap is cleaved by FEN1. To test this possibility, we examined LIG I activity on a nicked substrate in the presence of increasing concentrations (0–1000 nM) of the downstream strand of the damaged strand containing either a 5’-DOB (Fig 9A, left panel) or 5'-THF (Fig 9A, right panel). The amount of ligated product decreased significantly with increasing concentration of the downstream strand with a 5'-DOB (Fig 9A, left panel, compare lanes 3–8 with lane 2, percentage of ligated product is shown below each gel). However, the amount of ligated product was unaffected by the addition of the same concentrations of the corresponding oligonucleotide containing a 5'-THF (Fig 9A, right panel compare lanes 11–16 with lane 10, and quantification of ligated product shown below each gel). This indicates that the oxidized abasic lesion, DOB at the 5'-end of the downstream strand, inhibited LIG I, presumably by crosslinking the ligase. Using borohydride to trap any DNA-protein crosslinks, we observed a crosslink between DOB and LIG I by detecting a crosslink complex of a ssDNA-DOB-LIG I by SDS-PAGE under the same experimental conditions in which DOB inhibited LIG I (Fig 9B, lanes 8–10). The results showed that without photolysis, i.e. no DOB lesion, no protein-DNA complexes were detected (Fig 9B, lanes 2–5). With the DOB lesion, an ssDNA-DOB-LIG I crosslink complex was detected by the borohydride trapping assay (Fig 9B, lanes 8–10). No protein-DNA crosslink products were detected with the substrate alone or in the absence of borohydride with or without photolysis (Fig 9B, lanes 1 and 6, lanes 2 and 7). The results indicate that the single-strand flap with a DOB lesion cleaved by FEN1 inhibited LIG I by crosslinking to it, preventing completion of the repair process. Consequently, BER intermediates with DNA strand breaks accumulated. Since DOB-containing ssDNAs at 10–1000 nM significantly inhibited LIG I activity (Fig 9, panel A), whereas the same concentrations of THF-containing ssDNA (Fig 9, panel B) failed to do so, we propose that DOB does so specifically, rather than by general competitive binding of ssDNA to the enzyme.

## Discussion

In this study, for the first time, we identified the adverse effects of an oxidized abasic site DOB that is produced by antitumor agents on BER efficiency and TNR stability by inhibiting key BER enzymes. We found that DOB inhibited pol β synthesis activity (Figs 2 and 3) during BER in duplex and double-flap CAG repeat tracts by crosslinking with the polymerase (Fig 4A and 4B). DOB inhibited pol β and limited its insertion to ≤ 2 nucleotides. This resulted in accumulation of gapped intermediates generated by efficient FEN1 removal of a CAG repeat flap with a 5’-DOB. Subsequent formation of a ligatable nick that can be sealed by LIG I is also prevented, resulting in accumulation of unrepaired gapped BER intermediates. Surprisingly, we also found that DOB inhibited LIG I activity (Fig 9A) by crosslinking with the ligase (Fig 9B), and contributing to a reduction in BER efficiency (Figs 7 and 8). However, we found that the inhibition of BER key enzymes by the oxidized sugar did not affect the instability of CAG repeats during BER (Figs 7 and 8). Instead, it led to the accumulation of unrepaired BER intermediates (Figs 7 and 8). Our results support a mechanism by which DOB reduces BER efficiency by crosslinking with pol β and LIG I, thereby inactivating these enzymes, and leading to accumulation of strand break intermediates in TNR tracts. Through crosslinking with the lysines in the dRP lyase domain of pol β (Fig 4) [29], DOB inactivates the enzyme depleting the functional form of pol β and leading to an insufficiency of pol β and accumulation of gaps resulting from efficient FEN1 cleavage. This prevents the formation of a nick that can be sealed by LIG I. On the other hand, the activity of LIG I is inhibited presumably by the crosslink between the oxidized sugar at the 5'-end of a FEN1-cleaved flap and a key lysine residue in the catalytic site of LIG I [38, 39] that is involved in enzymatic catalysis (Fig 9). This would result in the accumulation of unrepaired nicked intermediates in CAG repeat tracts that may ultimately be subjected to double-strand DNA repair mediated by homologous recombination [40].

In this study, we found that although the dynamic nature of TNRs allows DNA slippage, creating a gap greater than 1 nucleotide that forces repair into the long-patch BER subpathway, regardless of the type of damage to the sugar residue [13] in either a duplex repeat tract or hairpin, the oxidized sugar, DOB appeared to be able to inhibit pol β activity by crosslinking with the polymerase. We speculate that this occurred when the DOB containing 5’-flap realigned to the template strand, positioning the DOB in a proximity to pol β at a nick or gap so that a crosslink formed with Lys72 or other lysine(s) in the lysine pocket of pol β dRP lyase domain [41], as these lysines are directly or indirectly involved in Schiff base formation during the β-elimination reaction [42]. The resulting DNA-protein crosslink irreversibly inhibits pol β dRP lyase activity and its synthesis of CAG repeats.

We also provide the first evidence that an oxidized abasic site, DOB, at the 5'-end of a single strand DNA that mimics a FEN1-cleaved flap inhibits the activity of LIG I by forming a covalent crosslink with the enzyme. This further suggests that similar to its reaction with K72 in the pol β dRP lyase domain, DOB reacts with one or more lysine residues in the catalytic site of LIG I. It has been found that Lys568 of LIG I is located adjacent to the phosphate group at the 5'-end of the nick, suggesting that this lysine residue plays a critical role in mediating the ligation reaction of the enzyme [39]. Crosslinking to Lys568 of LIG I by DOB inactivated the enzyme, resulting in the accumulation of unrepaired nicked intermediates in CAG repeat tracts.

Our results demonstrate that a DOB lesion decreases BER efficiency, causing the accumulation of DNA strand breaks during BER in TNR tracts, although it did not directly modulate the instability of CAG repeats during BER. We hypothesize that this is due to its inhibition of pol β and LIG I. This in turn prevented the formation of repaired products with repeat instability. It should be also noted that because of the length limitation for synthesis of the DOB-containing substrates, in this study, we only examined the formation of pol β-DOB crosslink complex on the substrates with a flap containing 2–5 CAG repeats. Our results do not rule out the possibility that the DOB lesion on a CAG repeat flap longer than 5 repeats directly modulate the instability of a long TNR tract by crosslinking with pol β and inhibiting the DNA synthesis activity of the enzyme.

A previous study has shown that another type of oxidized sugar, the C4-oxidized abasic site (C4-AP), which contains the same functional 1,4-dicarbonyl group as DOB crosslinks with the dRP lyase domain of pol β [43]. However, C4-AP inhibits pol β less efficiently than DOB (~7 turnovers compared to ~4 turnovers for DOB) because the ketone group at the C4 position is less reactive than the aldehyde in DOB. This decreased inactivation efficiency may allow sufficient gap-filling synthesis and strand displacement synthesis [43, 44] that in turn can promote TNR expansion. Thus, it is possible that C4-AP in a TNR tract may lead to detectable TNR instability by allowing more efficient BER and production of sufficient BER products with TNR instability. The roles of a C4-AP lesion in modulating TNR instability during BER remain to be elucidated.

In conclusion, in this study, we provide the first evidence that an oxidized abasic site, DOB, when present in CAG repeat tracts, inhibits pol β mediated DNA synthesis and LIG I activity through crosslinking with the enzymes during BER. This subsequently leads to inefficient BER, resulting in the accumulation of DNA strand breaks in TNR tracts. We suggest that this can further lead to error-prone DNA repair, such as DNA recombination, causing TNR instability.

## Supporting information

S1 File Supplementary methods. (PDF)

S1 Table Oligonucleotide sequences. (PDF)

S1 Fig Preparation of DOB-containing substrates via photolysis. DOB-containing substrates were prepared by exposure of o-nitrobenzyl protected lesions to 365 nm UV for 20 minutes.
(PDF)

S2 Fig Pol β DNA synthesis on the nicked and double-flap CAG repeat substrates containing a 5’-phosphate during BER. Pol β DNA synthesis activity on a nicked flap (left panel) and double-flap (right panel) with a 5’-phosphate was examined as described in the “Methods”. Lanes 1 and 7 indicate the substrate only. Lanes 2–6 and lanes 8–12 indicate the substrate incubated with pol β (2.5 nM) at the time interval of 2, 5, 10, 15, and 30 minutes. Substrates were ^32^P-labeled at the 5’-end of the upstream strand and are illustrated above each gel. The experiments were repeated at least three times, and only the representative gels were shown in the figures. Two-way ANOVA with Tukey’s multiple comparison posttests was used to determine statistically significant differences. "*" denotes P < 0.05, compared to the DOB-containing substrates.
(PDF)

S3 Fig Pol β crosslinked with DOB have lost its dRP lyase activity. Pol β dRP lyase activity was measured as described in S1 File. Lane 1 indicates the substrate containing a uracil only (25 nM). Lane 2 indicates the reaction with the substrate, 5 U UDG and 10 nM APE1. Lane 3 illustrates the reaction with the substrate, 5 U UDG and 10 nM APE1 in the presence of 340 mM NaBH_4_. Lane 4 illustrates the reaction with the substrate, 5 U UDG, 10 nM APE1 and 2.5 nM pol β without NaBH_4_. Lane 5 indicates the reaction with the substrate, 5U UDG, 10 nM APE1, 2.5 nM pol β and 340 mM NaBH_4_. Lane 6 indicates the reaction with the substrate, 5 U UDG, 10 nM APE1, and 2.5 nM pol β that was pre-incubated with the unphotolyzed nick-flap substrate in the presence of 340 mM NaBH_4_. Lane 7 illustrates the reaction with the substrate, 5 U UDG, 10 nM APE1 and 2.5 nM pol β that was pre-incubated with the photolyzed nick-flap substrate (pol β precrosslinked with DOB) in the presence of 340 mM NaBH_4_. Lane 8 indicates the reaction with the substrate, 5 U UDG, 10 nM APE1 and 2.5 nM pol β that was pre-incubated with the unphotolyzed double-flap substrate in the presence of 340 mM NaBH_4_. Lane 9 indicates the reaction with the substrate, 5 U UDG, 10 nM APE1 and 2.5 nM pol β that was pre-incubated with the photolyzed double-flap substrate (pol β precrosslinked with DOB) in the presence of 340 mM NaBH_4_. Substrates were ^32^P-labeled at the 3’-end of the damaged strand and are illustrated above each gel. The experiments were repeated at least in triplicate, and only the representative gel was shown in the figures. The quantification results were shown below the gel.
(PDF)
