# Supplementary material for: An oxidized abasic lesion inhibits base excision repair leading to DNA strand breaks in a trinucleotide repeat tract
Source: PLoS One. 2018 Feb 1;13(2):e0192148. doi: 10.1371/journal.pone.0192148 (PMC5794147; doi:10.1371/journal.pone.0192148)
Supplement: S1 File — (PDF) [file pone.0192148.s001.pdf]

S1 File. Supplementary methods.

Measurement of Pol β dRP lyase activity using dRP lyase activity assay

Pol β dRP lyase activity was examined in 10 µl-reaction mixture containing 30 mM HEPES, pH 7.8, 50 mM KCl, 0.5% inositol, 0.1 mg/ml BSA, 5 mM Mg^{2+} and 25 nM preincised P^{32}-labeled AP site containing substrates. An AP site was generated by pre-incubating the uracil-containing substrate (S1 Table) with 5 U UDG for 30 minutes. The photolyzed DOB-containing substrate was subjected to photolysis at 365 nm UV light for 20 minutes to remove the protecting group for creating the DOB lesion immediately prior to incubation with pol β at 37 °C for 30 min. The dRP lyase activity assay was initiated by adding 2.5 nM of pol β that was pre-crosslined with DOB-containing substrates and incubated at 37 °C for 30 min. The reaction was terminated by transferring reaction mixture on ice, and the dRP lyase products were stabilized by addition of 340 mM $NaBH_4$ and incubation on ice for 30 min. The dRP lyase activity of pol β that was incubated with the unphootylzed substrate was measured as the control of the experiments. The dRP lyase products were recovered by ethanol precipitation and resuspended in 10 µl of 2×stopping buffer. Substrates and products were separated by 18% urea-denaturing PAGE.
