# Supplementary material for: An oxidized abasic lesion inhibits base excision repair leading to DNA strand breaks in a trinucleotide repeat tract
Source: PLoS One. 2018 Feb 1;13(2):e0192148. doi: 10.1371/journal.pone.0192148 (PMC5794147; doi:10.1371/journal.pone.0192148)
Supplement: S1 Table — (PDF) [file pone.0192148.s002.pdf]

S1 Table. Oligonucleotide sequences

Oligonucleotides	nt	Sequence (5' -3')
Downstream/Damaged strand		
D1	32	**U** CAG CAG CAG CAG ATG CCC GCC CCA CTC ATT GC
D2	32	**F** CAG CAG CAG CAG ATG CCC GCC CCA CTC ATT GC
D3	32	**D** CAG CAG CAG CAG ATG CCC GCC CCA CTC ATT GC
D4	16	**p** CGT GCG GAT CCG GTG C
D5	32	**p** CAG CAG CAG CAG ATG CCC GCC CCA CTC ATT GC
D6	48	CTG GAT ACA CGA ACT TTA AGC A**U**A GTC AAT GAA GGA CGC ATA TCA GTG
Template strand		
T1	55	GCA ATG AGT GGG GCG GGC ATC TGC TGC TGC TGC TGT ACG GGC GCT AGG CGA CTC G
T2	46	GCA ATG AGT GGG GCG GGC ATC TGC TGT ACG GGC GCT AGG CGA CTC G
T3	31	GCA CCG GAT CCG CAC GGC GCA TCA GCT GCA G
T4	48	CAC TGA TAT GCG TCC TTC ATT GAC TCT GCT TAA AGT TCG TGT ATC CAG
Upstream strand		
U1	34	CGA GTC GCC TAG CGC CCG TAC AGC AGC AGC AGC A
U2	20	CGA GTC GCC TAG CGC CCG TA
U3	15	CTG CAG CTG ATG CGC

U: deoxyuridine
F: tetrahydrofuran, THF
D: 5'-(2-phosphoryl-1,4-dioxobutane), DOB
p: phosphate
