# Supplementary figures and images for: An oxidized abasic lesion inhibits base excision repair leading to DNA strand breaks in a trinucleotide repeat tract
Source: PLoS One. 2018 Feb 1;13(2):e0192148. doi: 10.1371/journal.pone.0192148 (PMC5794147; doi:10.1371/journal.pone.0192148)

S1 Fig

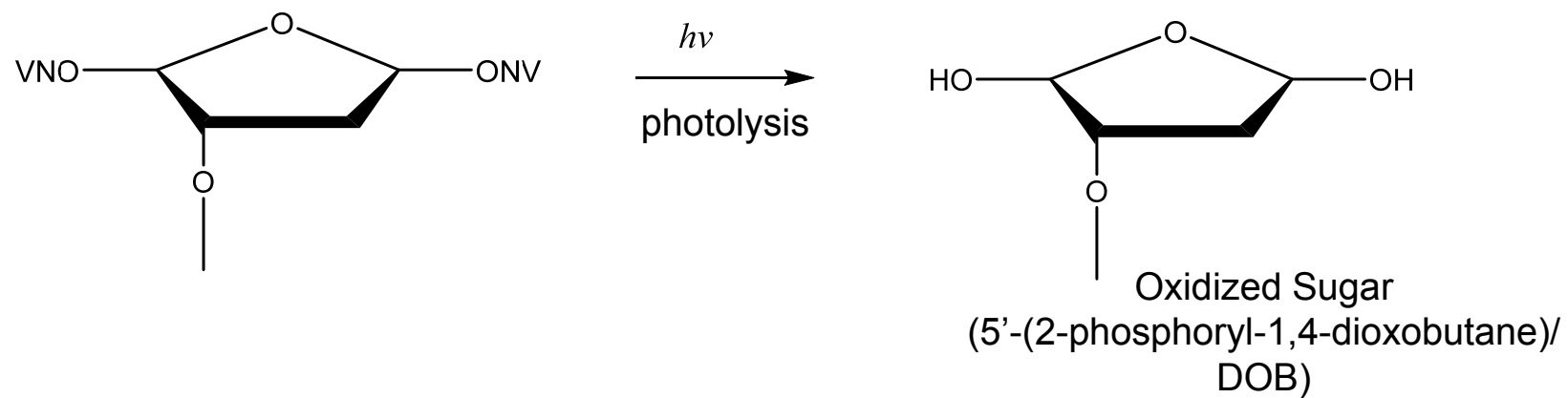

ONV= *o*-nitrobenzyl protecting group

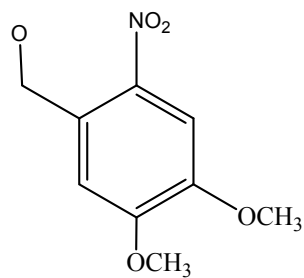

Supplement: S1 Fig — DOB-containing substrates were prepared by exposure of o-nitrobenzyl protected lesions to 365 nm UV for 20 minutes. (PDF) [file pone.0192148.s003.pdf]

**S2 Fig**

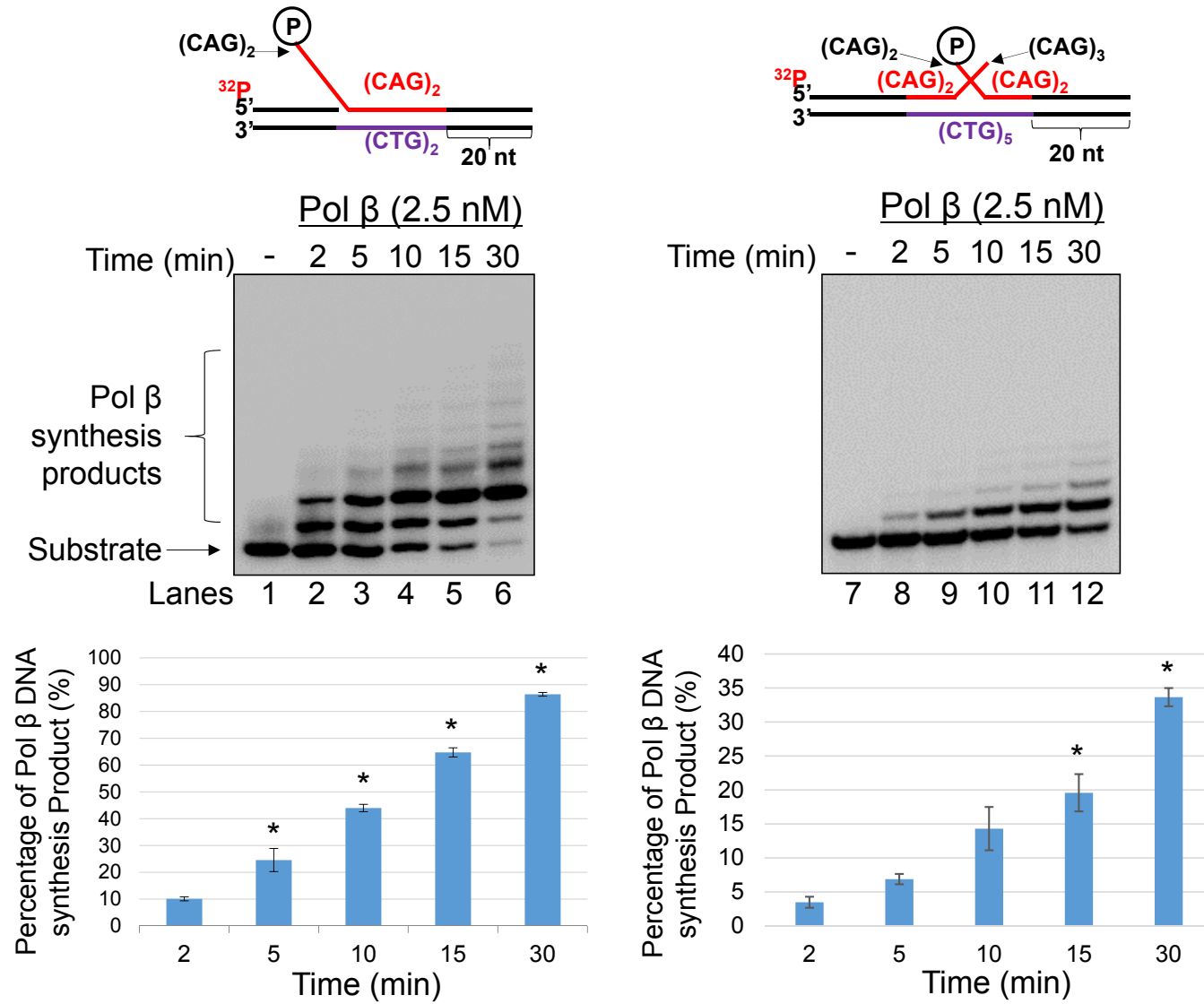

Supplement: S2 Fig — Pol β DNA synthesis activity on a nicked flap (left panel) and double-flap (right panel) with a 5’-phosphate was examined as described in the “Methods”. Lanes 1 and 7 indicate the substrate only. Lanes 2–6 and lanes 8–12 indicate the substrate incubated with pol β (2.5 nM) at the time interval of 2, 5, 10, 15, and 30 minutes. Substrates were 32P-labeled at the 5’-end of the upstream strand and are illustrated above each gel. The experiments were repeated at least three times, and only the representative gels were shown in the figures. Two-way ANOVA with Tukey’s multiple comparison posttests was used to determine statistically significant differences. "*" denotes P < 0.05, compared to the DOB-containing substrates. (PDF) [file pone.0192148.s004.pdf]

S3 Fig

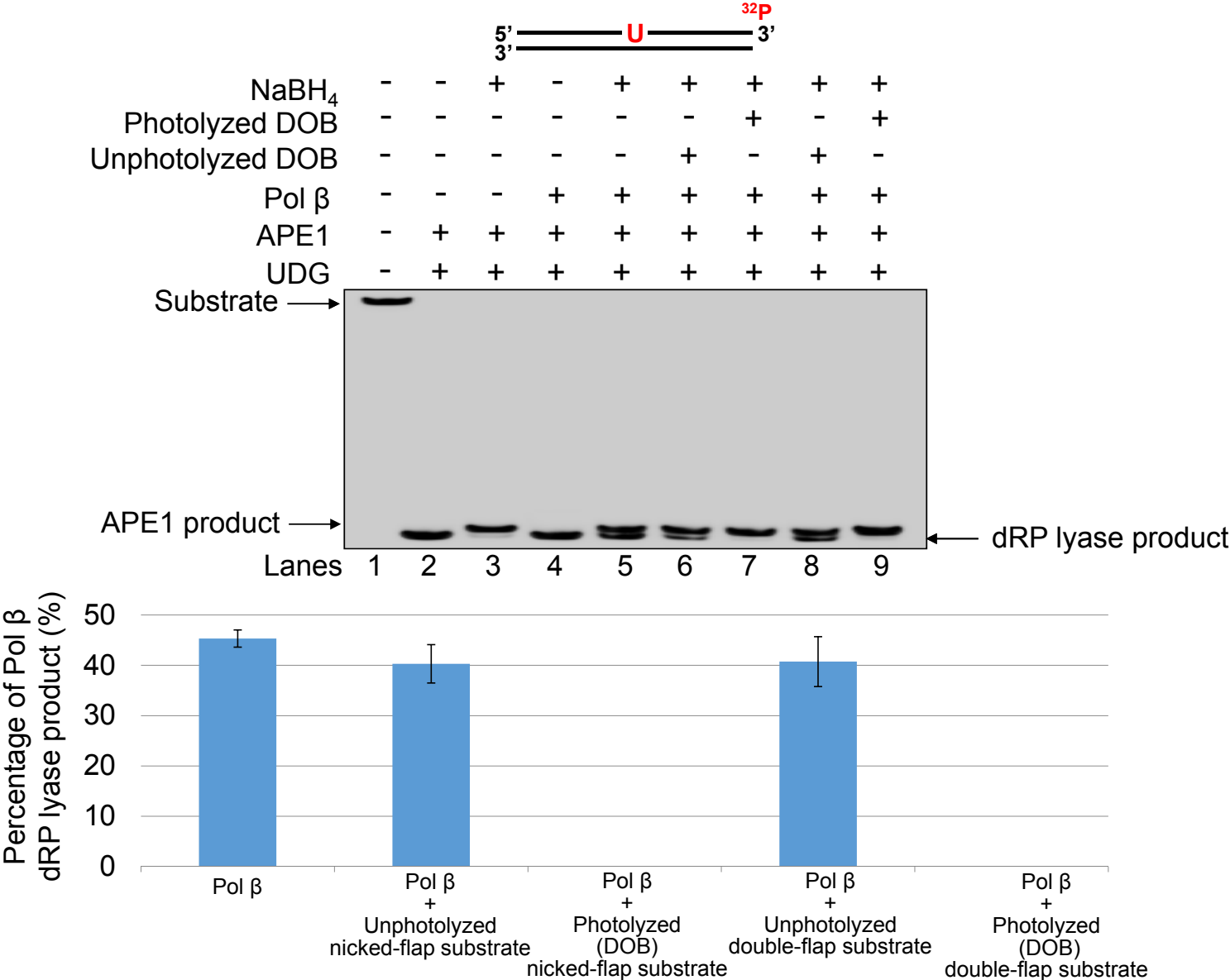

Supplement: S3 Fig — Pol β dRP lyase activity was measured as described in S1 File. Lane 1 indicates the substrate containing a uracil only (25 nM). Lane 2 indicates the reaction with the substrate, 5 U UDG and 10 nM APE1. Lane 3 illustrates the reaction with the substrate, 5 U UDG and 10 nM APE1 in the presence of 340 mM NaBH4. Lane 4 illustrates the reaction with the substrate, 5 U UDG, 10 nM APE1 and 2.5 nM pol β without NaBH4. Lane 5 indicates the reaction with the substrate, 5U UDG, 10 nM APE1, 2.5 nM pol β and 340 mM NaBH4. Lane 6 indicates the reaction with the substrate, 5 U UDG, 10 nM APE1, and 2.5 nM pol β that was pre-incubated with the unphotolyzed nick-flap substrate in the presence of 340 mM NaBH4. Lane 7 illustrates the reaction with the substrate, 5 U UDG, 10 nM APE1 and 2.5 nM pol β that was pre-incubated with the photolyzed nick-flap substrate (pol β precrosslinked with DOB) in the presence of 340 mM NaBH4. Lane 8 indicates the reaction with the substrate, 5 U UDG, 10 nM APE1 and 2.5 nM pol β that was pre-incubated with the unphotolyzed double-flap substrate in the presence of 340 mM NaBH4. Lane 9 indicates the reaction with the substrate, 5 U UDG, 10 nM APE1 and 2.5 nM pol β that was pre-incubated with the photolyzed double-flap substrate (pol β precrosslinked with DOB) in the presence of 340 mM NaBH4. Substrates were 32P-labeled at the 3’-end of the damaged strand and are illustrated above each gel. The experiments were repeated at least in triplicate, and only the representative gel was shown in the figures. The quantification results were shown below the gel. (PDF) [file pone.0192148.s005.pdf]
